# Rapid bone regeneration by *Escherichia coli*-derived recombinant human bone morphogenetic protein-2 loaded on a hydroxyapatite carrier in the rabbit calvarial defect model

**DOI:** 10.1186/s40824-015-0039-x

**Published:** 2015-07-16

**Authors:** Chung-Hoon Chung, You-Kyoung Kim, Jung-Seok Lee, Ui-Won Jung, Eun-Kyoung Pang, Seong-Ho Choi

**Affiliations:** Department of Periodontology, College of Dentistry, Yonsei University, 50 Yonsei-ro Seodaemun-gu, Seoul, 120-752 Republic of Korea; Department of Periodontology, School of Medicine, Ewha Womans University, Seoul, Republic of Korea

**Keywords:** Hydroxyapatite, *Escherichia coli*-derived recombinant human bone, Morphogenetic protein-2, Bone regeneration, Tissue engineering, Calvarial intraosseous defect model

## Abstract

**Background:**

The aim of this study was to determine the osteoconductivity of hydroxyapatite particles (HAP) as a carrier for *Escherichia coli*-derived recombinant human bone morphogenetic protein-2 (ErhBMP-2). Two 8-mm diameter bicortical calvarial defects were created in each of 20 rabbits. One of each pair of defects was randomly assigned to be filled with HAP only (HAP group) or ErhBMP-2 loaded HAP (ErhBMP-2/HAP group), while the other defect was left untreated (control group). The animals were killed after either 2 weeks (*n* = 10) or 8 weeks (*n* = 10) of healing, and histological, histomorphometric, and tomographic analyses were performed.

**Results:**

All experimental sites showed uneventful healing during the postoperative healing period. In both histomorphometric and tomographic analyses, the new bone area or volume of the ErhBMP-2/HAP group was significantly greater than that of the HAP and control groups at 2 weeks (*p* < 0.05). However, at 8 weeks, no significant difference in new bone area or volume was observed between the ErhBMP-2/HAP and HAP groups. The total augmented area or volume was not significantly different between the ErhBMP-2/HAP and HAP groups at 2 and 8 weeks.

**Conclusions:**

Combining ErhBMP-2 with HAP could significantly promote rapid initial new bone formation. Moreover, HAP graft could increase new bone formation and space maintenance, therefore it might be one of the effective carriers of ErhBMP-2.

## Background

Bone augmentation is generally carried out using autogenous bone, allograft, xenograft, or alloplastic materials. The ideal bone graft includes elements that are osteogenic, osteoinductive, and osteoconductive. Autogenous bone contains all three types of elements, but it is not available in every situation. Many studies have shown that xenograft or alloplastic materials augmented with growth factors improve bone regeneration, a major focus of tissue engineering. Numerous growth factors that enhance various types of cell migration, adherence, and proliferation have been identified. One of these, bone morphogenetic protein (BMP), is a multifunctional protein with a wide range of biological activities in a variety of cell types [1]. BMPs regulate growth, differentiation, chemotaxis, and apoptosis. They also play pivotal roles in morphogenesis [2]. BMPs constitute the osteoinductive component of several tissue engineering products that are used in late-stage development as replacements for autogenous bone grafts and for bone augmentation and repair [3]. Many studies support the use of recombinant human BMP-2 (rhBMP-2) [4, 5]. However, BMP-2 derived from Chinese hamster ovary cells (CHO BMP-2) is relatively costly because protein yields are low. In this context, our research group recently succeeded in producing BMP-2 using an *Escherichia coli* production system (ErhBMP-2), which is particularly attractive for biotechnology because of the ability of *E. coli* to grow rapidly and to high density on inexpensive substrates [6]. Moreover, ErhBMP-2 and CHO BMP-2 may function similarly in bone regeneration [7]. Several studies have demonstrated the efficacy of ErhBMP-2; for example, it was shown that ErhBMP-2 facilitated closure of the bone gap of a sinus window [8] and ErhBMP-2-coated implants enhanced bone-to-implant contact [9]. Various carriers have been recommended, including fibrin-fibronectin, biphasic calcium phosphate, beta-tricalcium phosphate (β-TCP), and hydroxyapatite (HA) [10]. The biological response to bone substitute materials depends not only on their chemical composition but also on their macro- and microstructural characteristics, including pore size, porosity, and interconnectivity [11]. The United States Food and Drug Administration has approved the use of BMP with an absorbable collagen sponge as carrier. However, ErhBMP-2 can be separated from the collagen under physical pressure, and collagen is rapidly absorbed. The carrier needs to be able to maintain space for subsequent bone formation. One carrier with this ability is HA. HA is used as a bone graft extender for posterolateral spinal fusion in humans [12]. It is also useful as an ErhBMP-2 carrier because of its high affinity for ErhBMP-2. ErhBMP-2-adsorbed hydroxyapatite particles (HAP) are safe and can be an effective and attractive material for bone formation, since the pore size of HAP is approximately 100–300 μm. The optimal pore size for bone regeneration is known to be 300–400 μm. The minimal interconnection pore size is 5–15 μm for fibrous tissue, 40–100 μm for osteoid tissue, and 100 μm for mineralized bone. Therefore, it appears that the pore size of HAP is suitable for promoting early bone ingrowth. Furthermore, in another study, alkaline phosphatase activity was significantly higher in mandibular defects treated with porous HA and ErhBMP-2 than in controls treated with HA alone at both 7 and 21 days [13], indicating that ErhBMP-2 accelerated bone formation by osteoconduction from porous HA.

β-TCP is more bioresorbable than HAP and is replaced by new bone at a high rate [14]. In a study that compared changes in the distribution and expression of biomarkers of reactogenicity in the lower jaws of rabbits after implantation, osteoblast proliferation and regions of granulation tissue formation were more noticeable in experimental tissues than in the control tissue. The experimental and control groups did not differ significantly in mean β-defensin-2, IL-1, IL-6, IL-8, IL-10, osteopontin, osteocalcin, BMP-2/4, or osteoprotegerin expression. Furthermore, the prevalence of osteopontin- and osteocalcin-positive osteocytes in experimental tissues implanted with HAP at 3 months after implantation indicated potential bone regeneration stimulated by pure HAP. Therefore, the slow resorption of HAP may enhance osteoconductivity, thereby promoting new bone growth. Based on these studies, we aimed to evaluate the effect of HAP on bone regeneration and to determine the efficacy of HAP as a carrier for ErhBMP-2 in the rabbit calvarial intraosseous defect model.

## Methods

### Animals

Twenty male New Zealand white rabbits (age, 9–20 months; body weight, 3–3.5 kg) were used in this study. The animals were housed in divided cages under standard laboratory conditions and fed a standard diet. The selection of experimental animals, their management, and the surgical protocol followed routines approved by the Institutional Animal Care and Use Committee of Yonsei Medical Center, Seoul, Korea.

### Materials

Large amounts of BMP-2 are difficult to purify or produce in vitro using eukaryotic cells. Human recombinant BMP-2 produced in *E. coli* is a homodimeric, non-glycosylated polypeptide containing 2 × 115 amino acids, with a molecular mass of 26 kDa. The ErhBMP-2 used in this study was provided by Daewoong Pharmaceutical Co., Ltd. (Novosis®-dent, Gyeonggi, South Korea). Lyophilized BMP-2 was dissolved in 10 cm^3^ of distilled water to yield a concentration of 0.1 mg/mL. HAP, manufactured by BioAlpha Inc. (Bongros®, Gyeonggi, South Korea), was used as the carrier material. Bongros® is composed of pure HAP and has a particle diameter of 0.6–1.0 mm. Bongros® was loaded with ErhBMP-2 by soaking 0.1 g of the material in 0.15 mL of ErhBMP-2 solution for 10 min. An ErhBMP-2 dose of 1.5 μg was achieved.

### Study design

Two circular calvarial intraosseous defects (8 mm in external diameter) were created side by side. Rabbits were divided into two treatment groups: (1) HAP only and (2) ErhBMP-2-loaded HAP (*n* = 10 animals per group). In each animal, graft materials were grafted into one of the defects, while the other defect was designated a sham surgery control and was filled with blood clots alone. The experimental sites for introduction of HAP or ErhBMP-2-loaded HAP were randomly allocated. The surgeon was not informed of the allocation until the defects had been created.

### Surgical protocol

**Fig. 1:**
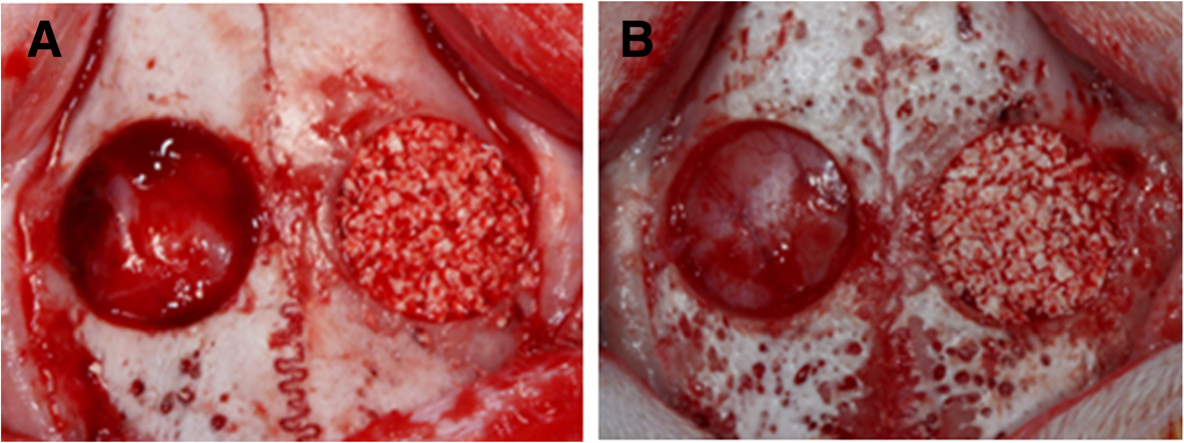
Two circular intraosseous defects of 8 mm diameter were made in each rabbit calvarium. The experimental graft materials (**a**, ErhBMP-2/HAP; **b**, HAP only) were grafted into one defect, and the other defect was left untreated as a control

Rabbits were anesthetized with an intramuscular injection of a 4:1 solution of ketamine hydrochloride (Ketalar, Yuhan, Seoul, Korea) and xylazine (Rompun, Bayer Korea, Seoul, Korea). The surgical site was shaved and disinfected with povidone iodine, and then infiltration anesthesia was induced by injection of 2 % lidocaine (lidocaine-HCl, Huons, Seoul, Korea). An incision was made in the sagittal plane, and a full-thickness flap was elevated. The two circular defects were then created in each animal using 8-mm trephines under cool saline irrigation. The distance between the defects was 3 mm (Fig. 1). The assigned graft material was grafted into one of the defects. The soft tissue was repositioned and then sutured layer-by-layer using 4–0 synthetic absorbable multifilament suture materials (VicrylPlus Antibacterial, Ethicon, Somerville, NJ, USA). Postoperative antibiotics (gentamicin; 5 mg/kg body weight) were administered by daily intramuscular injection for 1 week. The rabbits were killed at either 2 weeks (*n* = 5 per group) or 8 weeks (*n* = 5 per group) post-surgery.

### Histological processing

Blocks that included the adjacent tissues were harvested. The blocks were fixed in 10 % buffered formalin for 10 days, decalcified in 5 % formic acid for 14 days, and then embedded in paraffin. Serial sections of 5-μm thickness were cut. The two center-most sections were selected from each block and stained with hematoxylin and eosin.

### Evaluation methods

#### Clinical observation

Animals were carefully observed for inflammation, allergic reactions, and other complications surrounding the surgical site throughout the 2- and 8-week postoperative healing periods.

#### Histological observation

Specimens were examined under a microscope (DM LB, Leica Microsystems, Wetzlar, Germany) equipped with a camera (DC300F, Leica Microsystems) by a single, blinded examiner. Images of the slides were acquired and saved as digital files. Sections were examined at a magnification of 40 × .

#### Histomorphometric analysis

**Fig. 2:**
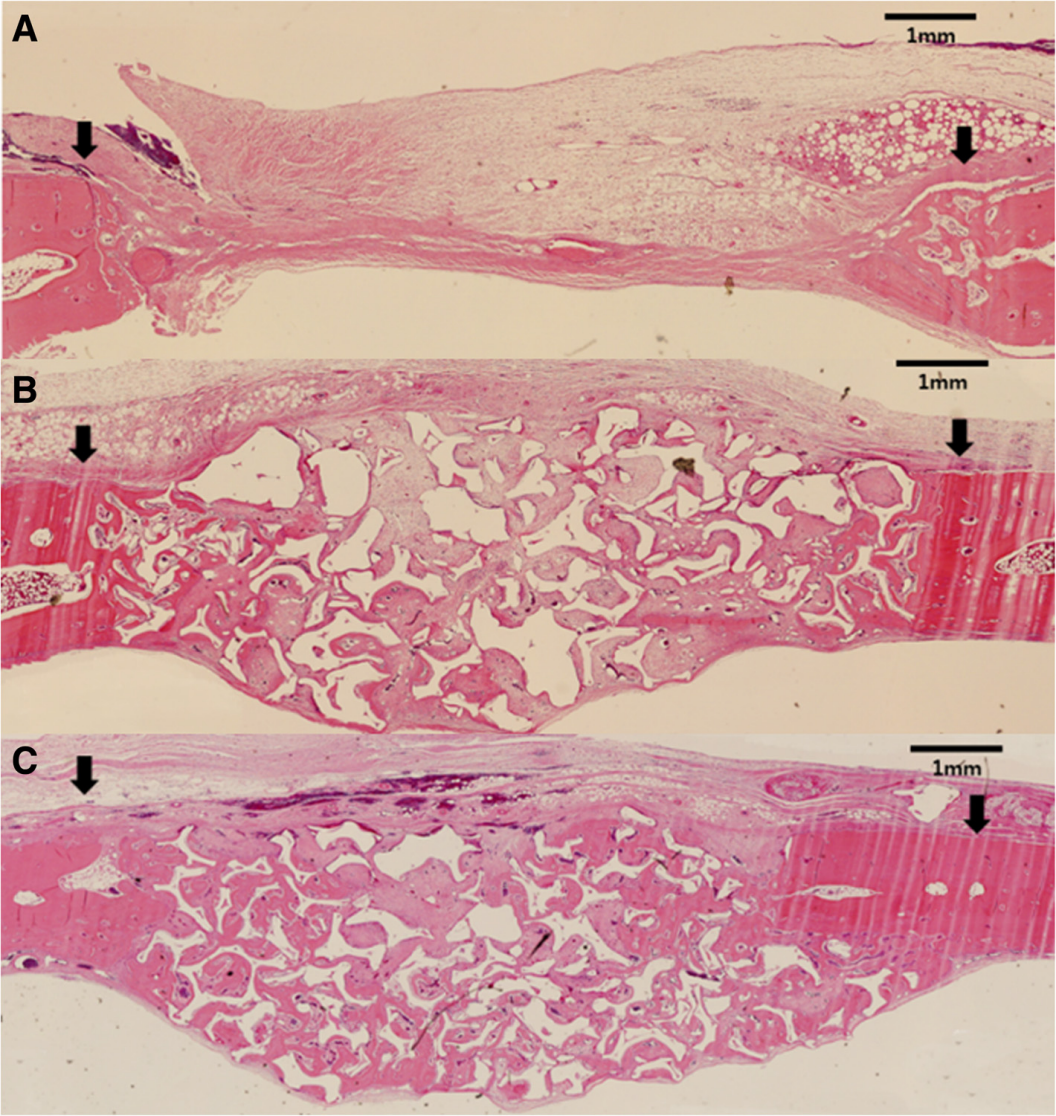
Representative photomicrographs obtained at 2 weeks postoperation. **a** Control, **b** HAP only, **c** ErhBMP-2/HAP (hematoxylin and eosin, ×40). Arrowheads = defect margin

**Fig. 3:**
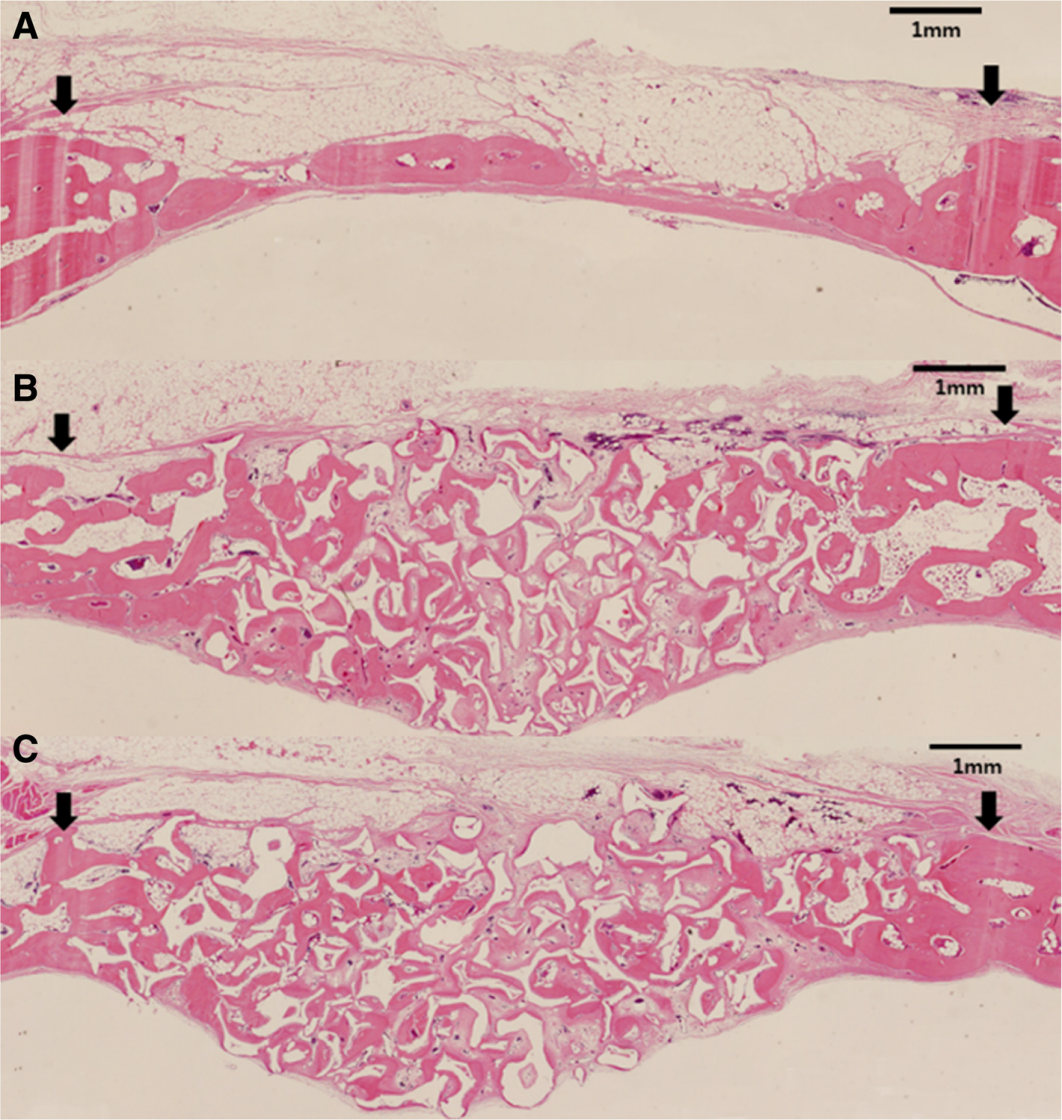
Representative photomicrographs obtained at 8 weeks postoperation. **a** Control, **b** HAP only, **c** ErhBMP-2/HAP (hematoxylin and eosin, ×40). Arrowheads = defect margin

Histomorphometric data for the following parameters were obtained with an automated image-analysis system (Image-Pro Plus, Media Cybernetics, Silver Spring, MD, USA; Figs. 2 and 3):

1. Total augmented area (mm^2^): the area of all tissues between the defect margins, including new bone, connective tissue, and vessels
2. New bone area (mm^2^): the area of newly formed bone within the total augmented area; and
3. Residual particle area (mm^2^): the area of HAP remaining within the defect.

#### Tomographic analysis

**Fig. 4:**
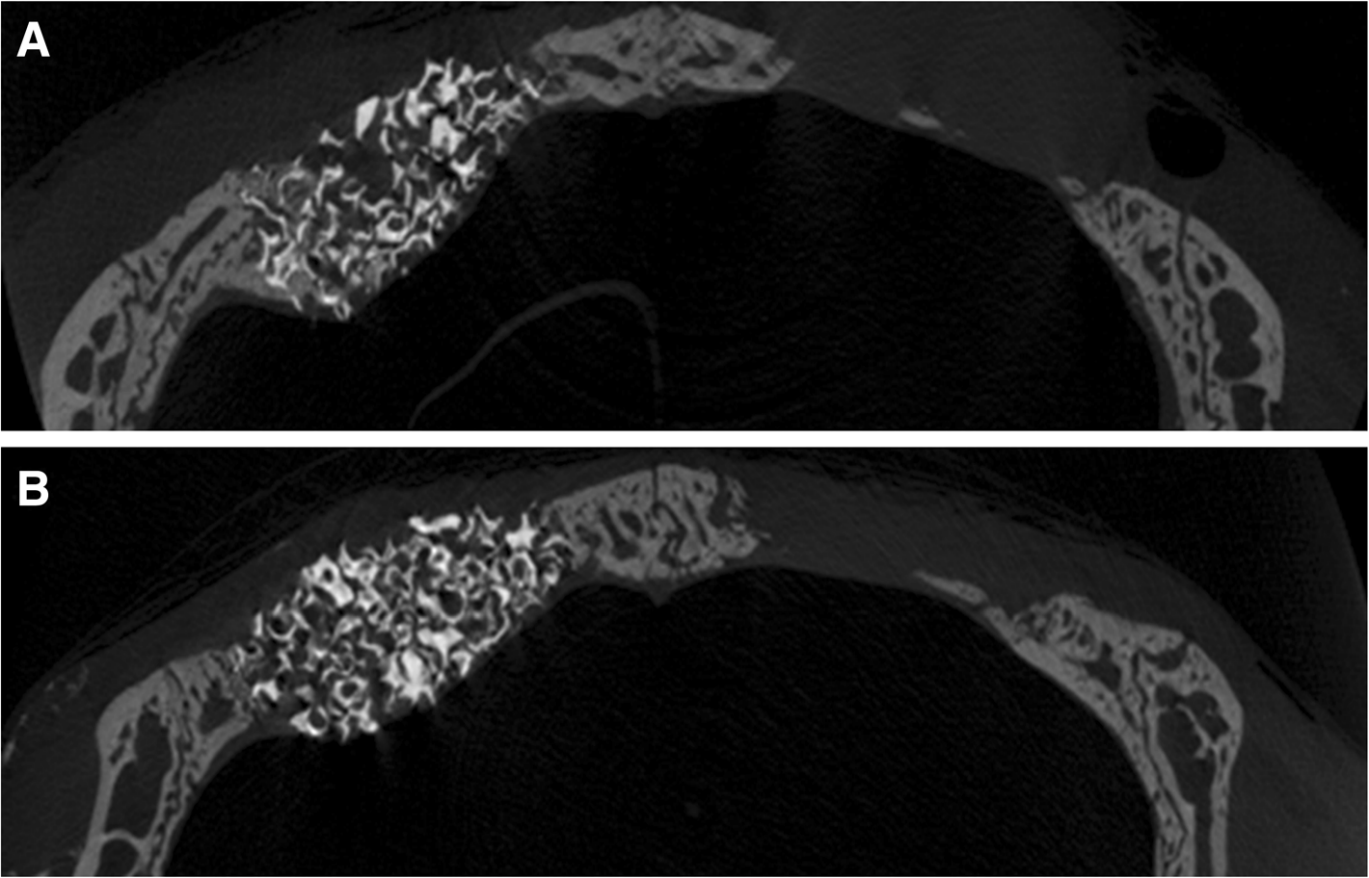
Representative coronally sectioned micro-computed tomography images at 2 weeks postoperation. **a** ErhBMP-2/HAP group, **b** HA only group

**Fig. 5:**
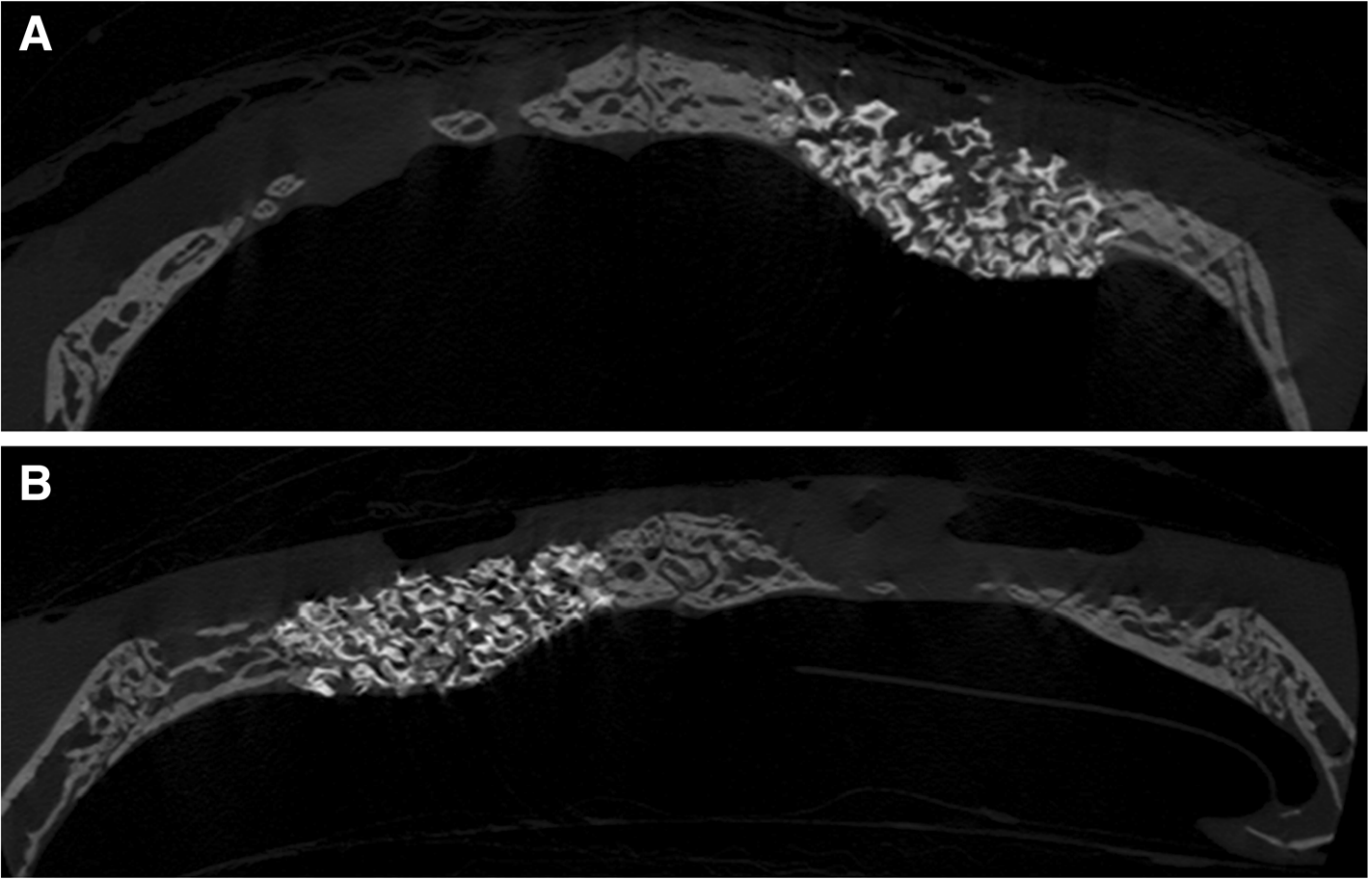
Representative coronally sectioned micro-computed tomography images at 8 weeks postoperation. **a** ErhBMP-2/HAP group, **b** HA only group

Specimens were scanned using a microcomputed tomography (micro-CT) system (SkyScans1072, SkyScan, Aartselaar, Belgium) at a resolution of 18 μm (100 kV and 100 μA) (Figs. 4 and 5). The scanned sets of data were processed in DICOM format, and the sum of the cross-sectional view was used to reconstruct the area of interest [15]. The overall dimensional topography of the recipient beds was measured in the reconstructed views.

#### Statistical analysis

Statistical analysis was performed using a commercially available software program (SPSS 15.0, SPSS, Chicago, IL, USA). Data from the histological and three-dimensional micro-CT sections are presented as mean ± standard deviation. The Kruskal–Wallis test was used to compare the control, HAP only, and ErhBMP-2-loaded HAP groups. The Mann–Whitney *U* test was used to compare samples collected at 2 weeks and 8 weeks post-surgery. The level for statistical significance was set at *p* < 0.05.

## Results

### Clinical findings

All experimental sites showed uneventful healing during the postoperative healing period. No evidence of complications, such as abnormal bleeding, infection, or exposure of graft materials, was observed. Signs of inflammation, such as swelling, were minimal, and the grafted materials were confirmed to be intact within the defects at the time of sacrifice and sample collection.

### Histological findings

After 2 weeks of healing, the sham surgery control defects in the HAP only and ErhBMP-2/HAP groups showed a small amount of wedge-shaped new bone formation, limited to the defect margin. The amount of newly formed bone in defects with grafted material was greater in the ErhBMP-2/HAP group than in the HAP only group. The center of control defects was depressed, and thus flattened, by surrounding connective tissue and dura mater. In contrast, the center of HAP only and ErhBMP-2/HAP defects was elevated by the grafted material. New bone was formed at the defect margins (Fig. 2). After 8 weeks of healing, the HAP only and ErhBMP-2/HAP groups showed similar amounts of newly formed bone (Fig. 3). More newly formed bone was generated in the center of the defects during the 8-week healing period than during the 2-week healing period. The residual particle area was not reduced after 8 weeks of healing compared with 2 weeks of healing (Figs. 2 and 3).

### Histomorphometric findings

**Fig. 6:**
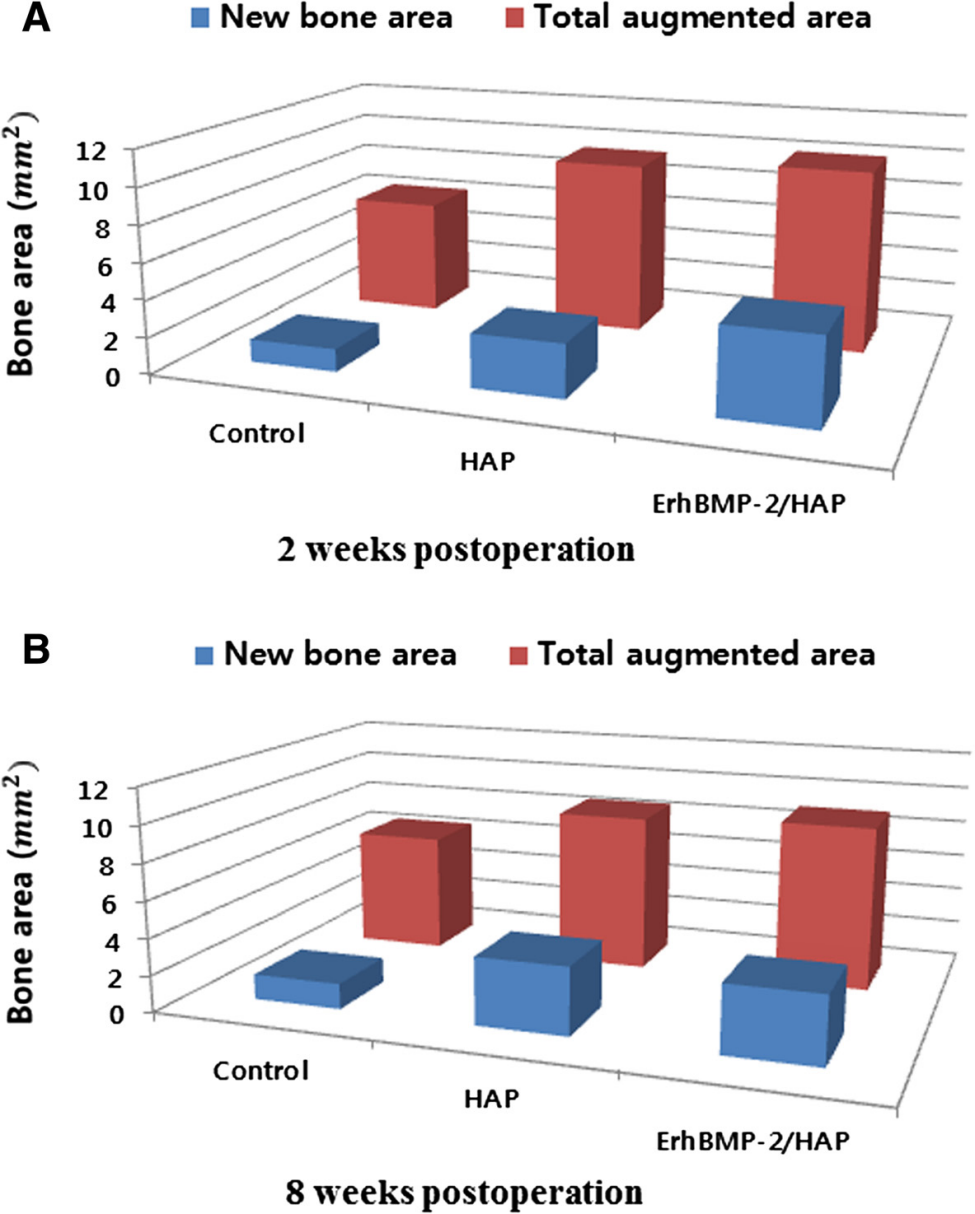
Graphs showing histomorphometric analysis of total augmented area and new bone area (mm^2^). **a** Two weeks postoperation, **b** 8 weeks postoperation. NA, new bone area; TA, total bone area

The histomorphometric measurements are summarized in Tables 1, 2, and 3. The total augmented area was significantly greater in the HAP only and ErhBMP-2/HAP defects than in controls at 2 and 8 weeks (Fig. 6). Within each group, no significant difference in total augmented area was observed between 2 and 8 weeks (Table 1). At 2 weeks, the area of new bone differed significantly between the HAP only and ErhBMP-2/HAP defects (Table 2). The amount of residual material was similar between HAP only and ErhBMP-2/HAP defects at 2 and 8 weeks (Table 3).

**Table 1:** Total augmented area of each group, as measured by histomorphometric analysis

Total augmented area (mm^2^)	2 weeks (*n* = 10)	8 weeks (*n* = 10)
Control	6.34 ± 0.17	6.54 ± 0.32
HAP	9.51 ± 0.61^*^	8.64 ± 0.38^*^
ErhBMP-2/HAP	10.05 ± 0.52^*^	9.02 ± 0.55^*^

Values are means ± standard deviation; *n* = number of specimens

*Significant difference compared with control group (*p* < 0.05)

**Table 2:** New bone area of each group, as measured by histomorphometric analysis

New bone area (mm^2^)	2 weeks (*n* = 10)	8 weeks (*n* = 10)
Control	1.25 ± 0.09	1.39 ± 0.13
HAP	2.94 ± 0.28^*^	3.68 ± 0.24^*^
ErhBMP-2/HAP	4.75 ± 0.50^*†^	3.67 ± 0.19^*^

Values are means ± standard deviation; *n* = number of specimens

*Significant difference compared with control group (*p* < 0.05)

†Significant difference between HAP and ErhBMP-2/HAP groups (*p* < 0.05)

**Table 3:** Residual particle area of each group, as measured by histomorphometric analysis

Residual particle area (mm^2^)	2 weeks (*n* = 10)	8 weeks (*n* = 10)
Control	NA	NA
HAP	1.99 ± 0.14	1.91 ± 0.38
ErhBMP-2/HAP	1.90 ± 0.34	1.82 ± 0.23

Values are means ± standard deviation; *n* = number of specimens

### Tomographic analysis

**Fig. 7:**
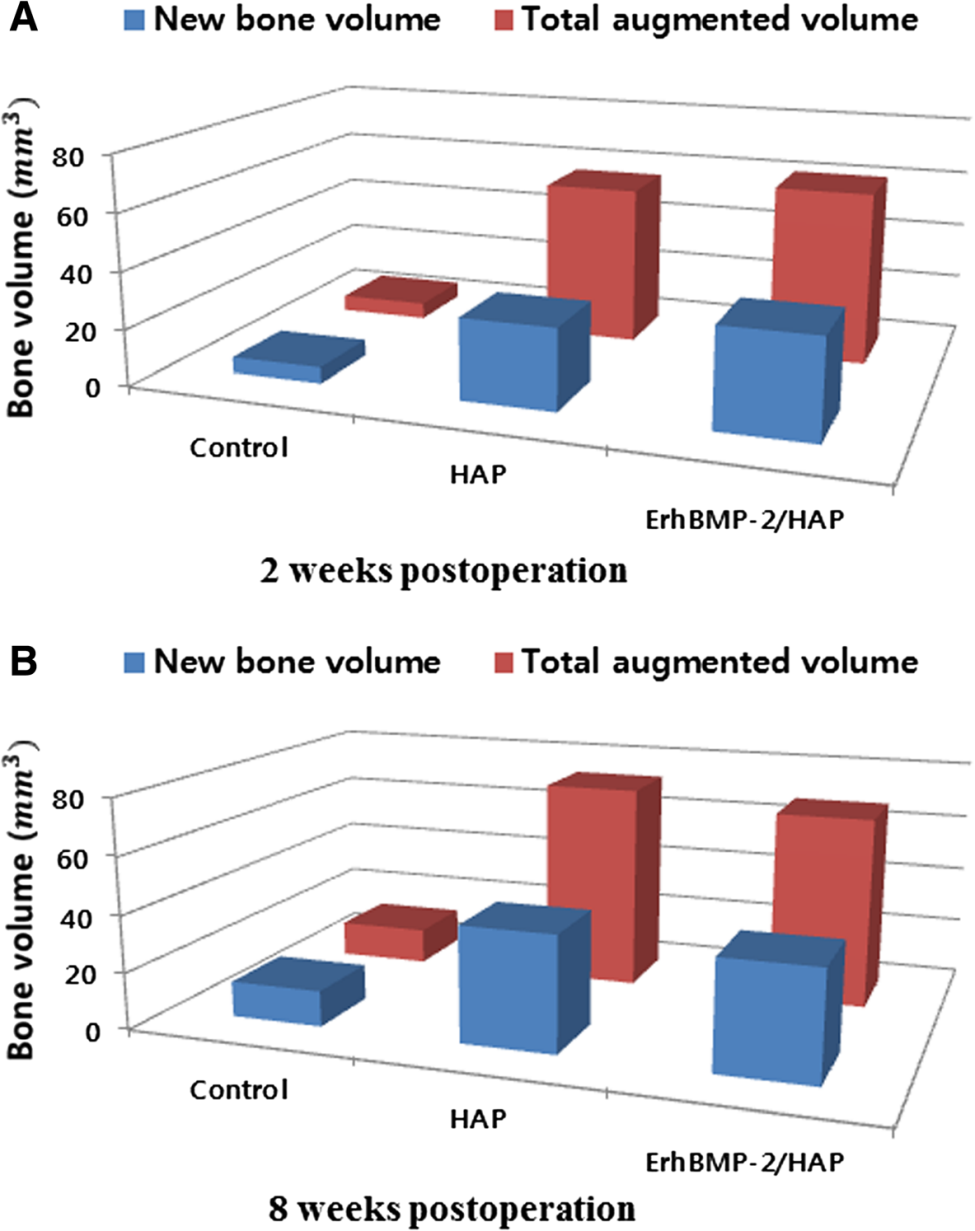
Graphs showing tomographic analysis of total augmented volume and new bone volume (mm^3^). **a** Two weeks postoperation, **b** 8 weeks postoperation. NV, new bone volume; TV, total bone volume

The overall dimensional topography of the defects and grafts was measured in reconstructed views at 2 and 8 weeks (Figs. 4 and 5). Newly formed bone was gray, while HAP was white because of radiopacity. The total augmented volume and new bone volume of each group were measured using micro-CT (Tables 4 and 5). At 2 and 8 weeks, the total augmented volume was significantly greater in the HAP only and ErhBMP-2/HAP groups than in controls (Fig. 7). However, the total augmented volume did not differ significantly between the HAP only and ErhBMP-2/HAP groups at either 2 or 8 weeks (Table 4). At 2 and 8 weeks, the new bone volume was significantly greater in the HAP only and ErhBMP-2/HAP groups than in controls (Fig. 7). At 2 weeks, the new bone volume was significantly greater in the ErhBMP-2/HAP group than in the other groups. At 8 weeks, the difference in new bone volume between the HAP only and ErhBMP-2/HAP groups was not significant (Table 5).

**Table 4:** Total augmented volume of each group, as measured by tomographic analysis

Total augmented volume (mm^3^)	2 weeks (*n* = 10)	8 weeks (*n* = 10)
Control	5.78 ± 0.54	12.61 ± 1.16
HAP	56.31 ± 2.62^*^	72.66 ± 4.07^*^
ErhBMP-2/HAP	61.12 ± 1.84^*^	67.55 ± 5.48^*^

Values are means ± standard deviation; *n* = number of specimens

*Significant difference compared with control group (*p* < 0.05)

**Table 5:** New bone volume of each group, as measured by tomographic analysis

New bone volume (mm^3^)	2 weeks (*n* = 10)	8 weeks (*n* = 10)
Control	5.78 ± 0.54	12.61 ± 1.16
HAP	28.40 ± 2.05^*^	40.60 ± 2.82^*^
ErhBMP-2/HAP	34.57 ± 1.65^*†^	38.31 ± 3.34^*^

Values are means ± standard deviation; *n* = number of specimens

*Significant difference compared with control group (*p* < 0.05)

†Significant difference between HAP and ErhBMP-2/HAP groups (*p* < 0.05)

## Discussion

BMP is a key factor in bone regeneration and healing. CHO BMP-2 is relatively expensive because of low production volumes. ErhBMP-2 is particularly attractive for biotechnology because of the ability of *E. coli* to grow rapidly and at high density on inexpensive substrates. Recombinant DNA techniques have been used to produce BMP-2 as an alternative to autograft bone to enhance healing of intraosseous defects.

Many studies have been performed to assess potential rhBMP-2 carriers. Either platelet-rich plasma or calcium phosphate can be used as a carrier of rhBMP-2 [16, 17]. The efficacy of an absorbable collagen sponge has also been demonstrated [18, 19]. The Infuse® system (Medtronic, Memphis, TN, USA) consists of rhBMP-2 on an absorbable collagen sponge carrier. OP-1® (Stryker Biotech, Kalamazoo, MI, USA) consists of rhBMP-7 and bovine collagen that has been reconstituted with saline to form a paste. However, collagen is not able to maintain space, which is crucial for excluding unwanted cells. For space maintenance during wound healing, biphasic calcium phosphate with a high proportion of HA may be a more appropriate rhBMP-2 carrier [20]. Bioactive glass fabricated with dicalcium phosphate dehydrate is not suitable as a BMP-2 carrier; a previous study showed that the bone mineral density, bone area, and bone mineral content of tibiae and contralateral femurs did not differ between control and BMP-treated groups [21]. The goals of this study were to evaluate the effect of HAP on bone regeneration and to determine the efficacy of HAP as a carrier for ErhBMP-2 in a rabbit calvarial intraosseous defect model.

Collagen carriers do not resist collapse caused by soft tissue pressure during bone formation. An investigation of the bone cell response to titanium surfaces showed that bone cell activities were enhanced in the presence of a BMP–atelopeptide type I collagen mixture [22]. Another study examined the effects of a BMP–atelopeptide type I collagen mixture on bond strength at the interface between bone and titanium implants. At 3 weeks post-surgery, the reverse torque of the BMP-treated group (74.2 ± 5.2 N · cm) was significantly greater than the reverse torque of the untreated group (32.8 ± 1.1 N · cm). At 12 weeks post-surgery, the difference between the reverse torque of the BMP-treated group (89.2 ± 2.7 N · cm) and that of the untreated group (75.8 ± 2.4 N · cm), although still statistically significant, was much smaller [23]. These results are concordant with the results of the present study, suggesting that the soaked carriers released the ErhBMP-2 early; this is a major limitation of currently available carriers.

Our histomorphometric analyses showed that the HAP only and ErhBMP-2/HAP groups had significantly larger areas of new bone than the control group at 2 weeks post-surgery. The ErhBMP-2/HAP group also differed significantly in new bone area from the control and HAP groups. Surprisingly, although the ErhBMP-2/HAP group showed a larger area of new bone than the HAP only group at 2 weeks post-surgery, no significant difference in new bone area was observed between the ErhBMP-2/HAP and HAP only groups at 8 weeks post-surgery. Thus, it can be inferred that the use of ErhBMP-2 with HAP as the carrier promoted rapid initial bone regeneration. According to Zhu et al. [24], the ability to repair bone defects decreases with time, although Nano-HA/rhBMP-2 composite artificial bone shows a good ability to repair bone defects.

Micro-CT images showed that the new bone volume of the ErhBMP-2/HAP group was significantly larger than that of the other groups at 2 weeks post-surgery. However, the ErhBMP-2/HAP and HAP only groups did not differ significantly in new bone volume at 8 weeks post-surgery. Consistent with this result, an in vitro study showed that in the initial period of cultivation and up to 72 h, coating of HAP with type I collagen had positive effects on the viability and osteoblastic characteristics of osteoblastic cells [25]. Therefore, it can be deduced that when guided bone regeneration is clinically required, HAP soaked in ErhBMP-2 can be applied without a membrane, since HAP can promote rapid initial bone generation. This technique would be easier than guided bone regeneration using an absorbable or non-absorbable membrane and could provide quick and easy promotion of rapid initial bone generation.

The total augmented area and volume did not differ between the HAP only and ErhBMP-2/HAP groups at 8 weeks post-surgery. In addition, the residual particle area did not differ significantly between 2 weeks and 8 weeks post-surgery. This indicates that HAP can maintain rigidity over a long period. The porous structure of HAP facilitated the infiltration and adherence of responsive cells, and the carrier itself became a component of the newly formed bone. HAP is osteoconductive and can maintain its original biocompatible form. Because of these qualities, HAP may be useful in augmentation of ridges and elevation of sinuses in clinical settings.

According to Lee et al. [26], there is a strong positive correlation between a high concentration of rhBMP and soft tissue swelling. It has been shown that the inflammatory response prompted by rhBMP lasts for only a short period. Although it varies according to volume, the degree of inflammation gradually decreases over the first 7 days; the authors therefore advise careful observation for 7 days after surgery. In our experiment, no side effects, such as seroma or edema, were observed for up to 8 weeks. This indicates that the amount of ErhBMP-2 used in this experiment was not high enough to cause serious side effects.

ErhBMP-2 is known to induce ectopic bone growth [27]. If control and ErhBMP-treated bone defects are too close together, ErhBMP-2 might flow into the control defect and cause unwanted bone regeneration, seroma, or edema [28]. In this study, no specific evidence of complications was observed. In a previous study, doubts were raised over whether a distance of 2 mm was sufficient to prevent control calvarial defects from being affected by ErhBMP-2 in neighboring defects [29]. In this study, a distance of 3 mm between the treated and control defects proved to be sufficient to allow comparison of healing responses. Therefore, ErhBMP-2-loaded HAP can be used as a graft material that does not affect nearby defects.

Although new bone volume in the ErhBMP-2/HAP group was rapidly promoted in the short term, it did not increase over the long term. This may be due to the limitations of HA as a carrier. According to Crouzier et al. [30], ErhBMP-2 adsorbed onto polyelectrolyte multilayer-coated films and, to a lesser extent, bare granules could be stored and remained bioactive for over 3 weeks. The in vivo release kinetics of BMP-2 from calcium-deficient hydroxyapatite (CDHA) scaffolds resembled the in vitro kinetics [31]. Similar observations have been made in other ectopic and orthotopic animal models [32]. Quantitative real-time PCR and enzyme-linked immunosorbent assay demonstrated that a lyophilized BMP-2/CDHA construct with trehalose (lyo-tre-BMP-2) significantly promoted osteogenic differentiation of bone marrow stromal cells [33]. The release rate of BMP-2 is critical to bone regeneration. BMP-2 was nearly 100 % released from lyo-tre-BMP-2 over 28 days. Adsorption of BMP-2 onto HA follows the Langmuir isotherm [34]. HAP may have more adsorption sites for its high specific surface area than HA block bone. Therefore, HAP may provide more opportunities for binding of ErhBMP-2 molecules. To develop an effective carrier, a method to release ErhBMP-2 from HAP at a consistent rate is required. Once this problem is solved, long-term increases in the volume and area of bone regeneration are expected to be realized. In future work, we will attempt to develop a method for slow, consistent release of ErhBMP-2 during long-term healing. In addition, other carriers, such as β-TCP, should be analyzed and compared.

## Conclusions

Combining ErhBMP-2 with HAP could significantly promote rapid initial new bone formation. Moreover, HAP graft could increase new bone formation and space maintenance, therefore it might be one of the effective carriers of ErhBMP-2. Furthermore, in the future, the identification of methods for slow and consistent release of ErhBMP-2 during long-term healing will be needed.
